# Longitudinal Improvements in Treatment Delivery Efficiency for MR-Guided Radiation Therapy: An 8-Year Single-Institution Experience

**DOI:** 10.3390/cancers18172814

**Published:** 2026-08-31

**Authors:** Robert A. Herrera, Sirikorn Unsri, Kathryn E. Mittauer, Rupesh Kotecha, Adeel Kaiser, Matthew D. Hall, Yongsook C. Lee, Yonatan Weiss, Tatiana Bejarano, Eyub Y. Akdemir, Mattison J. Flakus, Nikolai Strusberg-Fernandez, Ranjini Tolakanahalli, Nema Bassiri, Maria Ayala, Noah S. Kalman, Diane Alvarez, Tino Romaguera, Minesh P. Mehta, Alonso N. Gutierrez, Michael D. Chuong

**Affiliations:** 1 Department of Radiation Oncology, Herbert Wertheim Cancer Institute, Baptist Health South Florida, 8900 N. Kendall Drive, Miami, FL 33176, USA; 2 Department of Oncological Sciences, Herbert Wertheim College of Medicine, Florida International University, Miami, FL 33199, USA; 3 Department of Radiation Oncology, University of Texas San Antonio, San Antonio, TX 78249, USA

**Keywords:** MRgRT, MR-guided radiotherapy, stereotactic ablative radiotherapy, online adaptive radiotherapy, stereotactic body radiotherapy, SBRT, SMART, treatment time analysis

## Abstract

**Purpose**: While magnetic resonance-guided radiation therapy (MRgRT) may provide significant clinical advantages, delivery times tend to be longer than other radiation therapy (RT) modalities and published data on these times are limited. We evaluated longitudinal treatment times across our 8-year institutional MRgRT experience. **Methods/Materials**: A retrospective analysis of patients treated at our institution on a 0.35-Tesla MR-Linac between April 2018 and April 2026 was performed. All fractions were delivered with continuous intrafraction cine-MRI, soft tissue tracking, automatic beam gating, and online adaptive radiation therapy (oART) when indicated. The primary objective was to characterize changes in total in-room time (TIRT), treatment delivery time (TDT), and total adaptive time (TAT). For analysis of efficiency gains, our overall experience was separated into early (2018–2022) and late (2022–2026) periods. **Results**: A total of 1026 patients, 1203 treatment courses, and 7665 fractions were included. The median age was 69 years (range: 19–94) and the most commonly treated sites by treatment course were pancreas (n = 430; 35.7%), thorax (n = 203; 16.9%), abdominopelvic lymph nodes (n = 181; 15.0%), liver (n = 138; 11.5%), and adrenal gland (n = 83; 6.9%). The median prescription dose was 50 Gy (range: 16.0–76.0) in a median of five fractions (range: 1–36). Breath-hold and stereotactic body radiation therapy (SBRT) were used in 87.9% and 88.5% of treatment courses, respectively. From the early (2018–2022) to late (2022–2026) study period, the proportion of fractions utilizing SBRT (49.9% vs. 81.1%; *p* < 0.001), oART (32.9% vs. 80.2%; *p* < 0.001), respiratory gating (75.9% vs. 88.8%; *p* < 0.001), and elective nodal coverage (61.8% vs. 78.2%; *p* < 0.001) increased, as did the proportion of pancreatic treatments (20.4% vs. 38.4%; *p* < 0.001) and the utilization of single-fraction courses (3.4% vs. 10.3%; *p* < 0.001). Despite this increasing complexity between study periods, median oART TIRT decreased from 67.0 to 48.0 min (28.4% reduction; *p* < 0.001), driven primarily by a reduction in TAT from 20.0 to 7.0 min, *p* < 0.001. Pancreatic oART fractions showed the greatest improvement (median TIRT, 70.0 to 47.0 min; *p* < 0.001). In 2022–2026, 78.1% of oART fractions were completed within 60 min vs. 35.5% in 2018–2022 (*p* < 0.001). **Conclusions**: Ablative MRgRT, with or without oART, can often be delivered in 60 min or less, including for mobile and anatomically unfavorable tumors. Future software and hardware advances are expected to further improve MRgRT treatment efficiency.

## Simple Summary

Magnetic resonance-guided radiation therapy is an advanced cancer treatment that uses real-time imaging to precisely target tumors and spare nearby healthy organs. Treatment times are longer with this online adaptive radiation approach and this, to some extent, has limited its wider adoption. This study evaluated how treatment times changed over an 8-year period at a single high-volume center that treated over 1000 patients, predominantly with tumors in the pancreas and other abdominal sites. Despite increasingly complex cases over time, treatment delivery efficiency improved substantially, with the time required for adaptive treatments decreasing by nearly 30%, driven primarily by faster plan adaptation workflows, staff experience, and system upgrades enabling parallel team workflows. In 2022–2026, approximately 80% of adaptive treatments were completed within 60 min, including single-fraction courses. These findings demonstrate that this technology can be delivered within practical timeframes and may help guide other centers seeking to establish or optimize similar programs.

## 1. Introduction

Magnetic resonance-guided radiation therapy (MRgRT) features superior soft tissue contrast, real-time soft tissue tracking coupled with automatic beam gating, and online adaptive radiation therapy (oART) capabilities to aid radiotherapy deliveries [1,2]. This novel technology can facilitate safe dose escalation, reduced toxicity, and shorter fractionation schedules, particularly for mobile and anatomically unfavorable tumors near critical organs [3,4,5,6,7].

Although initially explored in the late 1990s, MRgRT has only entered routine clinical use over the past decade. Commercially available 0.35-T and 1.5-T MR-linac systems integrate onboard MR-imaging with daily online adaptive replanning [8,9]. Clinical indications have become better defined in recent years, particularly for mobile targets adjacent to dose-limiting organs at risk [10,11].

Although the clinical advantages of MRgRT are increasingly recognized, concerns regarding potentially prolonged treatment delivery times are a barrier to broader adoption [12]. MRgRT treatment duration is influenced by multiple factors including target location, intrafraction motion, use of oART, treatment platform characteristics (e.g., dose rate, beam gating), and overall workflow design [13]. Limited published data suggest that MRgRT delivery time requirements can vary greatly across institutions, which may reflect differences in treatment platforms, patient characteristics, target lesions, and institutional experience [14,15,16,17]. There is a need to minimize MRgRT treatment times to improve patient tolerability, optimize treatment unit throughput, and reduce treatment start delays. MR-guided SBRT has been modeled as cost-effective despite higher per-course costs, with treatment-unit time representing an important contributor to overall resource use [18,19,20,21]. Reducing per-fraction delivery time may therefore improve patient tolerability, increase treatment capacity, and enhance resource efficiency.

Our institution was an early MRgRT adopter starting in April 2018, and has since primarily treated with dose-escalated stereotactic body radiation therapy (SBRT) and oART [22]. Given the limited published data describing MRgRT treatment delivery times, we evaluated our 8-year institutional MRgRT experience to characterize longitudinal changes in delivery times and identify factors associated with treatment duration.

## 2. Methods and Materials

After obtaining institutional review board approval, a retrospective study of patients at our institution treated on a 0.35-T MRIdian MR-Linac (ViewRay, Oakwood Village, OH, USA) between April 2018 and April 2026 was performed. Patients who received MRgRT as a boost to the same lesion before or after non-MRgRT (e.g., CT-guided linac, proton therapy) within the same treatment course were excluded.

### 2.1. Treatment Planning

Our institutional MRgRT planning approach has been previously described [23,24]. Briefly, patients underwent MRI simulation in the MRIdian Linac (ViewRay, Oakwood Village, OH, USA) in addition to CT simulation, although in 2022 we transitioned to MRI-only simulation (using bulk density overrides to facilitate adequate dose calculation) for select abdominal targets. Patients with upper abdominal targets were instructed to fast for at least 2–3 h, as tolerated, prior to simulation and treatment. The gross tumor volume (GTV), clinical target volume (CTV, when indicated), and planning target volume (PTV) were defined with 3–5 mm isotropic setup margins, except for prostate lesions, in whom this 3–5 mm PTV expansion was not applied. CTV design typically consisted of a 2–5 mm isotropic expansion from the GTV, cropped out of natural barriers to spread with the exception of pancreatic cancer patients who increasingly were treated with an anatomically defined elective volume [25] and para-aortic nodal targets in which the elective volume may have included a ~1–2 cm region around the aorta. Fiducial markers and internal target volumes were not used. Treatment plans were optimized to achieve ≥95% PTV coverage at prescription dose, although under coverage was permitted if required to meet organ-at-risk (OAR) constraints. All treatment plans were developed using a step-and-shoot intensity-modulated RT (IMRT) technique. The number of beam angles and multi-leaf collimator (MLC) segments was optimized to reduce overall modulation and thereby shorten beam delivery time.

### 2.2. MRIdian Linac System

The MRIdian system pairs a 6-MV flattening-filter-free linear accelerator with a 0.35-T MRI scanner using a true fast imaging with steady-state precession (TrueFISP) sequence [26,27]. The linac delivers step-and-shoot IMRT with automatic beam gating driven by a single sagittal plane or multi-planar cine MRI and real-time tracking [28]. In addition to MR imaging, the system facilitates online adaptation of treatment plans [29,30,31]. The first oART treatment at our institution was delivered in September 2018.

The MRIdian system underwent two major upgrades during the study period. In April 2020, the 2.0 hardware and 5.3.0 software package introduced waveguide repositioning and faster MLC leaf speed. In July 2022, the A3i package (510(k) approved December 2021) added real-time multi-planar tracking, the BrainTx™ package, a gantry-specific homogeneity correction enabling 3D volumetric imaging at any gantry angle (prior versions required gantry rotation to a calibration angle of 330° for volumetric imaging), and a clinical parallel workflow that allowed multiple team members to perform tasks simultaneously during adaptive treatments. A3i further accelerated Monte Carlo optimization and dose calculation; removed the previous user-interface pauses; automatically initiated image acquisition upon radiofrequency-shielded door closure rather than requiring operator-triggered scan start; and enhanced the in-room monitor for improved visual feedback of gating during treatment delivery.

### 2.3. Treatment Delivery Workflow

Each treatment session began with alignment of the daily volumetric MR to the simulation reference MR scan. Prior to A3i, setup imaging consisted of two acquisitions: a free-breathing pilot scan for vertebral body alignment followed by a breath-hold volumetric scan. With A3i implementation, the free-breathing pilot scan was eliminated, reducing setup to a single breath-hold volumetric acquisition per fraction. The radiation therapist (RTT) performed any necessary adjustments under the supervision of the medical physicist (MP) and radiation oncologist (RO), who approved the final positioning registration. The necessity of oART was determined based on the similarity of OAR positioning relative to the simulation reference MR.

For oART, target volumes and OARs were recontoured daily to account for inter-fraction changes in anatomy. Previously contoured targets were rigidly registered to the daily MR scan, with modifications made only when the RO identified significant anatomical changes. All OAR contours were deformably registered from the simulation reference to the daily volumetric MR scan. The RO adjusted any OAR contours within 3 cm of the PTV in the axial plane (lateral and anterior/posterior directions) and 2 cm in the superior/inferior direction. The MP reviewed all contours and verified the bulk electron density override. The RTT contoured tracking structure(s) are to be used for real-time gating. After the A3i system upgrade, team members contoured simultaneously on separate workstations, enabling parallel contouring to reduce overall session time. Beginning in June 2021, in select cases, patients were placed on supplemental oxygen via nasal cannula to reduce respiratory motion, thereby narrowing the gating window and improving duty cycle efficiency during beam delivery.

Once recontouring was complete, the original treatment plan was mapped onto the anatomy of the day to visualize and analyze dosimetric coverage to targets and OARs. When needed, the MP re-optimized the plan, prioritizing OAR constraints before enhancing target coverage. The RO selected and approved the best available plan, which was then independently verified by the MP using a second Monte Carlo dose calculation with magnetic field correction. If the adapted plan did not result in a clinically meaningful improvement upon the original plan, then the original plan was delivered. Following plan selection prior to delivery, the RTT set the tracking plane and gating parameters with MP approval.

### 2.4. Treatment Time Definitions and Statistical Analysis

Timestamps for each workflow component of every delivered fraction were prospectively recorded by an RTT in an Excel spreadsheet beginning with our first patient treated in April 2018. Treatment time metrics were defined as follows:

- Setup and pre-treatment registration (setup initiation to registration completion);
- Total adaptive time (TAT; contouring, plan re-optimization, quality assurance, plan approval), as applicable;
- Treatment delivery time (TDT; initiation to completion of treatment beam delivery);
- Total in-room time (TIRT; patient entering room to treatment delivery completion, inclusive of TAT if oART was used).

Fractionation was classified as SBRT (≤6 fractions, ≥5 Gy per fraction), moderate hypofractionation (>6 fractions, >2 Gy per fraction), or conventional fractionation (1.8–2 Gy per fraction). Other regimens not meeting these specific criteria were palliative with different fractionation schemes. The biologically effective dose (BED_10_) was calculated using the linear-quadratic model with an α/β ratio of 10 Gy.

Statistical analyses were performed using Microsoft Excel 2022 (Microsoft Corporation) and SPSS version 27.0 (IBM). Continuous variables are reported as median with interquartile range (IQR) and categorical variables as frequency with percentage. Two-group comparisons used the Mann–Whitney U test. Categorical variables were compared using the chi-square test. All tests were two-sided with significance set at *p* < 0.05. To quantify efficiency gains with experience, the evaluated plans were separated into an early (2018–2022) and a late (2022–2026) cohort.

## 3. Results

From April 2018 to April 2026, 1026 patients received 1203 treatment courses consisting of 7665 fractions. Baseline demographics and treatment characteristics are summarized in Table 1, with most parameters being significantly different between early and late eras. Target lesions were most often located in the pancreas (n = 430; 35.7%), thorax (n = 203; 16.9%), abdominal or pelvic lymph node (n = 181; 15.0%), liver (n = 138; 11.5%), or adrenal gland (n = 83; 6.9%) (Supplemental Table S3). The median prescription dose was 50.0 Gy (range, 16.0–76.0 Gy) in a median of five fractions (range, 1–36 fractions); the median prescribed BED_10_ was 100.0 Gy_10_ (range, 28.0–200.0 Gy). SBRT was especially common (88.5% of all treatment courses) and included 89 single-fraction courses (median dose 34 Gy [range, 16–40]; median BED_10_ 149.6 Gy_10_). While automatic beam gating was used for all patients, 87.9% of courses were delivered using a breath-hold technique with a visual biofeedback system to improve duty cycle efficiency given the prevalence of mobile thoracic and abdominal target lesions.

**Figure 1:**
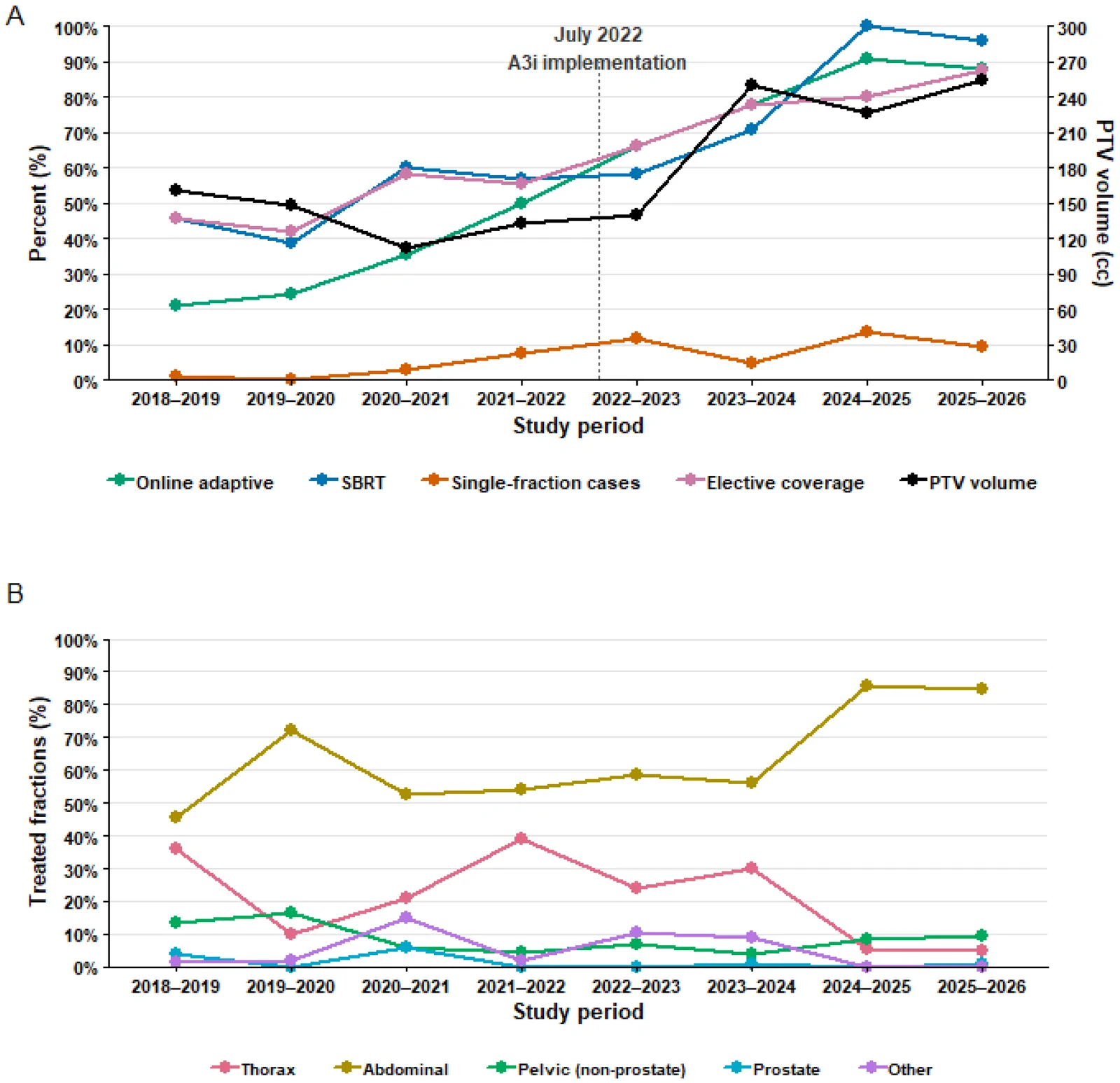
Proportion of total fractions delivered annually from 2018 to 2026 with stereotactic body radiation therapy (SBRT), online adaptive radiation therapy (oART), and for pancreatic cancer. Abbreviation: SBRT (stereotactic body radiotherapy); PTV (planning target volume). Panel (**A**,**B**) is distribution of treated fractions by disease site across study periods.

Over the 8-year study period, there were notable changes in MRgRT utilization that reflect an increase in treatment complexity (Figure 1). From the early (2018–2022) to late (2022–2026) era, the proportion of fractions utilizing SBRT (49.9% vs. 81.1%), oART (32.9% vs. 80.2%), elective coverage (61.8% vs. 78.2%), and pancreatic treatment (20.4% vs. 38.4%) all increased significantly (all *p* < 0.001) (Table 1). Median CTV and PTVs also increased over time, by 83.7% and 74.2%, respectively (CTV: 58.4 vs. 107.3 cc, *p* = 0.002; PTV: 133.9 vs. 233.3 cc, *p* < 0.001).

**Figure 2:**
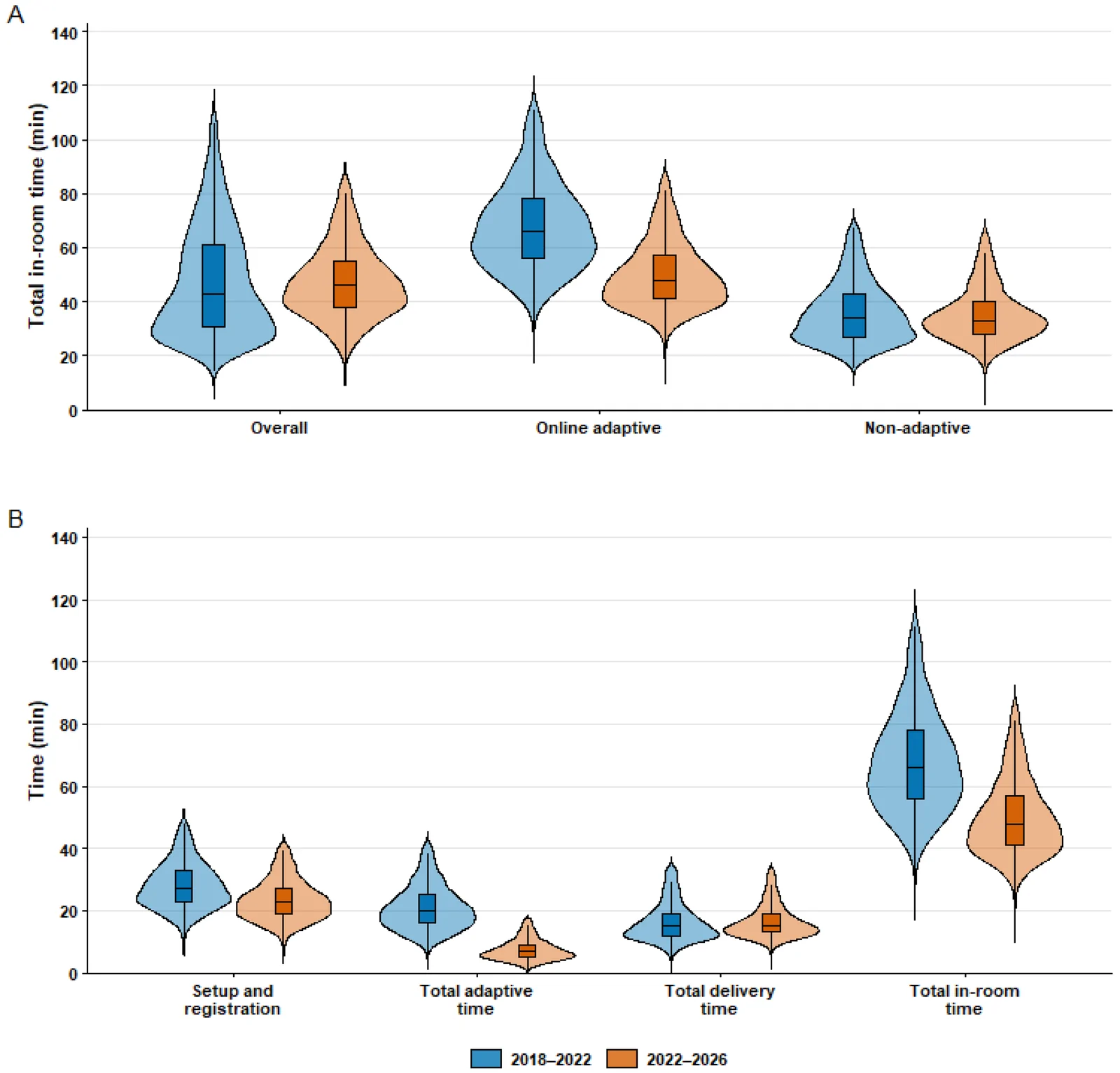
Median treatment times for the early (2018–2022) and late (2022–2026) study eras. Panel (**A**) shows total in-room time by workflow type and Panel (**B**) shows online adaptive workflow components.

Across all 7665 fractions, median TIRT was 45 min (IQR, 35–59 min), increasing from 43 to 46 min between the early and late eras (*p* < 0.001) (Figure 2). Of all evaluated treatment fractions, 76.3% (5849) were completed within 60 min, 61.2% (4690) within 50 min, and 50.6% (3876) within 45 min. The proportion of fractions completed within 60 min improved from 73.3% to 80.6% across eras (*p* < 0.001).

**Figure 3:**
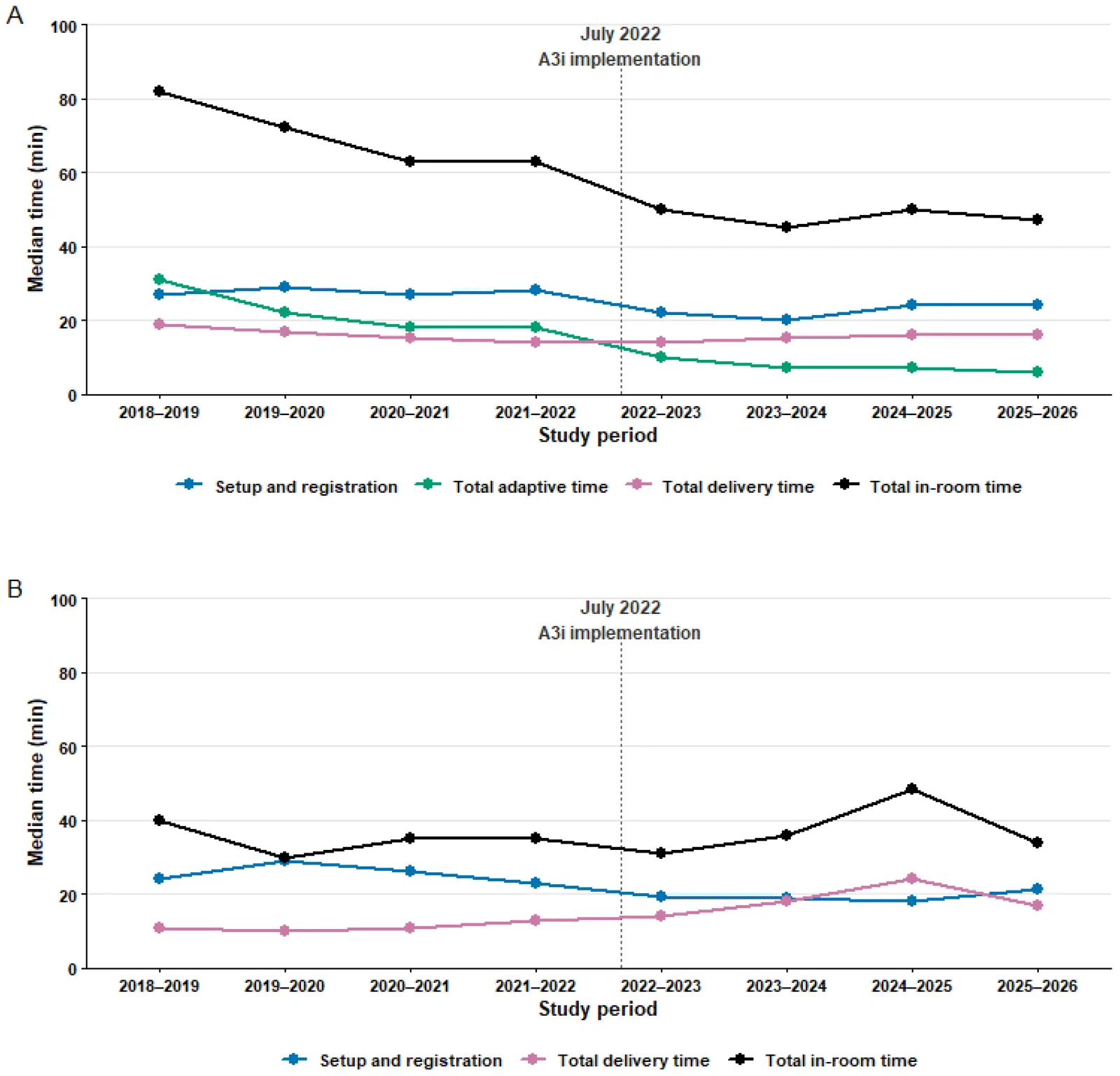
Treatment times by (**A**) online adaptive and (**B**) non-adaptive workflows across study periods.

Among 4277 oART fractions, median TIRT was 53.0 min overall and decreased from 67 min (IQR, 56–79 min) to 48 min (IQR, 41–59 min), a 28.4% reduction (*p* < 0.001) (Figure 3). Among all oART fractions, 64.8% (2771) were completed within 60 min, 43.5% (1860) within 50 min, and 30.0% (1278) within 45 min. The proportion of oART fractions completed within 60, 50, and 45 min improved from 35.5% to 78.1%, 13.0% to 57.2%, and 5.5% to 40.7%, respectively (all *p* < 0.001). Among oART fractions, the TIRT reduction was driven by reductions in TAT (20 to 7 min; *p* < 0.001) and setup time (28 to 23 min; *p* < 0.001), with a small increase in TDT (15 vs. 16 min; *p* = 0.012).

Among the 89 single-fraction SBRT courses, median TIRT was 66.0 min (IQR, 52.0–85.0) and median TDT was 38.0 min (IQR, 28.0–58.0). For oART single-fraction treatments (n = 38) that had a median prescribed BED_10_ of 87.5 Gy_10_, median setup and pre-treatment registration was 17.0 min (IQR, 15.0–23.2), TAT was 8.0 min (IQR, 6.0–10.2), TDT was 30.0 min (IQR, 25.0–42.0), and TIRT was 55.0 min (IQR, 50.0–71.0). Non-oART single-fraction treatments (n = 51) that had a median prescribed BED_10_ of 149.6 Gy_10_, median setup and pre-treatment registration was 19.0 min (IQR, 17.5–20.5), TDT was 46.5 min (IQR, 33.0–63.2), and TIRT was 71.5 min (IQR, 56.0–93.2). Supplemental material (Figures S1 and S2) further breaks down TIRT between single- and multi-fraction SBRT.

**Figure 4:**
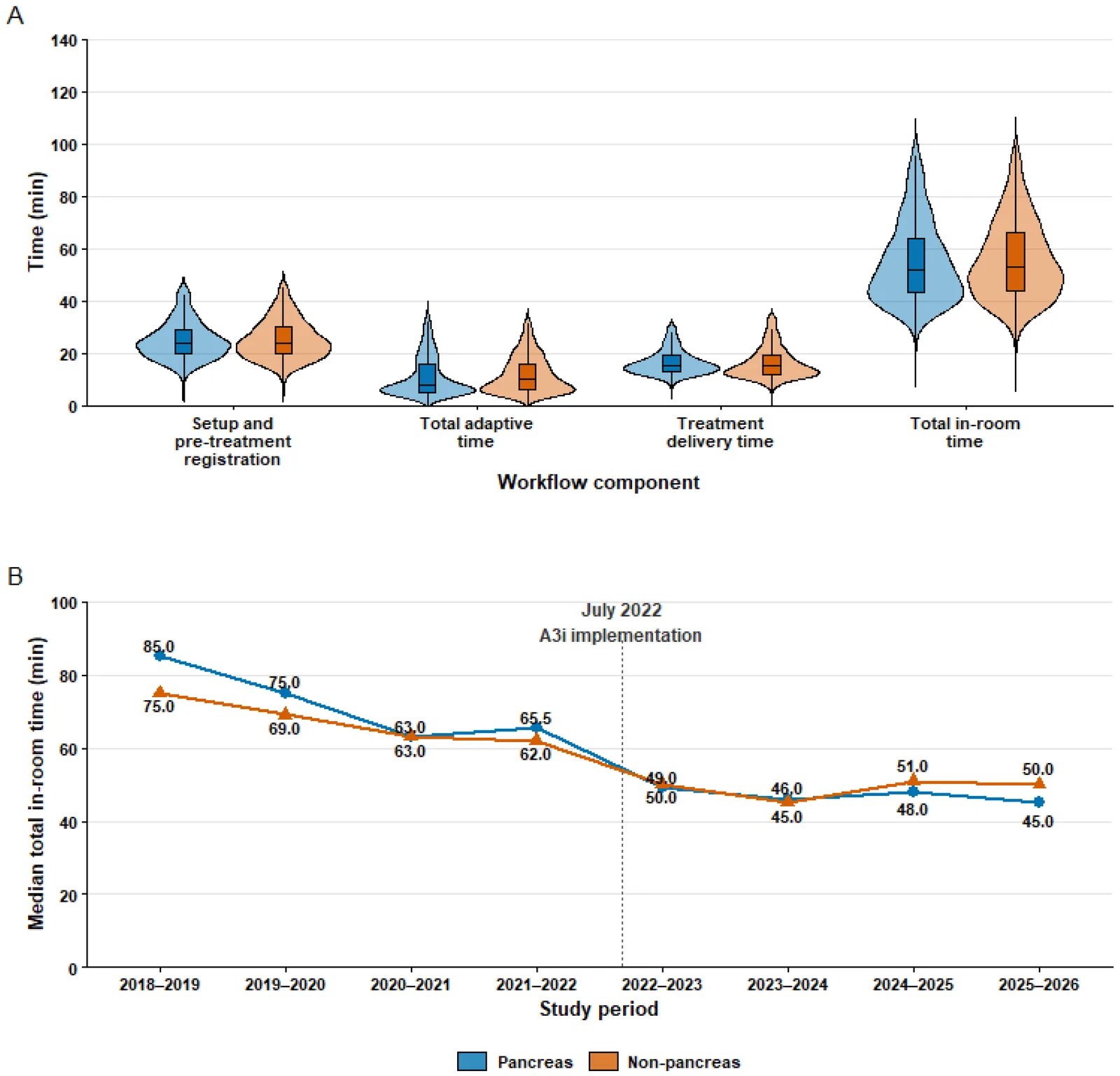
Median treatment times for the early (2018–2022) and late (2022–2026) study eras comparing pancreas and non-pancreas target lesions. Panel (**A**) shows online adaptive workflow components, and Panel (**B**) shows median in-room time for online adaptive fractions by annual study period for pancreas and non-pancreas targets.

Median pancreatic oART TIRT decreased from 85 to 45 min (47.1% reduction, *p* < 0.001) (Supplemental Table S1), compared with 75 to 50 min (33.3%, *p* < 0.001) for non-pancreatic sites. Early in the experience in 2018–2019, pancreas fractions were significantly longer than non-pancreas fractions (85 vs. 75 min in 2018–2019; *p* = 0.006); by 2025–2026, pancreas fractions were faster (45 vs. 50 min; *p* < 0.001) (Figure 4).

Among 3388 non-oART fractions, median TIRT was unchanged between eras (35 vs. 34 min; *p* = 0.447). The proportion completed within 60, 50, and 45 min remained stable at 91.6% vs. 90.4%, 84.0% vs. 83.9%, and 77.0% vs. 77.4%, respectively (all *p* > 0.05).

Among oART fractions, setup time decreased from 28 to 23 min (*p* < 0.001), TAT from 20 to 7 min (65.0% reduction; *p* < 0.001) and TDT was relatively stable (15 vs. 16 min; *p* = 0.012) (Figure 2). Among non-oART fractions, setup time decreased from 26 to 19 min (*p* < 0.001), while TDT increased from 11 to 16 min (*p* < 0.001), offsetting setup gains and resulting in unchanged TIRT (35 vs. 34 min; *p* = 0.447).

## 4. Discussion

To the best of our knowledge, this is one of the largest evaluations of MRgRT treatment times. Our findings are notable because median TIRT improved considerably over the first several years and remained low thereafter, despite a progressive shift toward more complex MRgRT treatment deliveries. By the final study period, nearly all patients were treated with ablative SBRT, most fractions incorporated oART, breath-hold gating was used across nearly all fractions, and pancreatic tumors treated with elective nodal coverage represented nearly half of all courses. This increasing complexity would typically be expected to increase treatment time, although median oART TIRT decreased to 47 min by 2025–2026 (Supplemental Table S2) and nearly 80% of oART fractions were completed within 60 min. Pancreatic oART demonstrated an even more striking reduction, with median TIRT decreasing from 85 to 45 min over the study period. These results suggest that in a mature high-volume program, gated oART MRgRT can be delivered within 50 min time slots even for complex mobile abdominal targets.

The decline in oART TIRT was primarily driven by significant reductions in setup and registration time as well as TAT. These time reductions are likely related to the increasing experience of our MRgRT program, along with deliberate efforts to standardize each component of the adaptive workflow so that quality and efficiency could be maintained even if different physicians, physicists, and RTTs were involved across treatment fractions. From the inception of our MRgRT program, the attending physician supervises all oART deliveries, which we believe contributes to both treatment efficiency and quality. We also increasingly prioritized advanced assignment of physician adaptive coverage during a weekly operations meeting to ensure timely availability for recontouring and plan approval needs.

The MRdian A3i upgrade in 2022 also contributed to reduced TAT, primarily through the introduction of a more efficient workflow in which the physician, physicist, and RTT could complete their individual adaptive tasks in parallel. Before this upgrade, we were limited to a serial oART workflow in which only one individual could work at any given time. Votta et al. published the first evaluation of the clinical parallel workflow enabled by the A3i upgrade across 254 fractions, estimating an approximately 25% reduction in TIRT compared with the serial workflow [16]. It is notable that our median TAT since 2022, despite increasing treatment complexities, was 7.0 min. TDT did not decrease over time, as expected, since our MR-Linac delivery characteristics (e.g., dose rate, step-and-shoot delivery) remained similar.

We believe that our results should challenge perceptions that developing a robust MRgRT program is impractical due to untenable time requirements. Johnstone et al. estimated that adaptive gated SBRT routinely requires approximately 90 min, significantly constraining daily capacity and financial viability [12]. In contrast, we demonstrate that TIRT for oART MRgRT is often half that time, in the ~45–50 min range, even for complex cases like pancreatic SBRT that require recontouring of not only the GTV but also a sizeable anatomically derived anisotropic CTV as well as close-proximity OARs [25]. Guckenberger et al. described MRgRT as limited by “increased treatment complexity, use of human resources, and longer treatment slot times, which translate to decreased throughput” [32]. While this characterization is partially accurate, it is important to recognize that the difference in gated MRgRT treatment time may not be substantially longer than motion-managed non-MRgRT for mobile abdominal targets. For example, non-adapted CyberKnife pancreas and liver SBRT series have reported treatment times of 40–50 min [33,34,35], non-adapted liver SBRT treated with breath-hold or gating may require ~40 min [36], and adapted CT-guided pancreas SBRT without elective coverage could require at least ~30 min [37,38]. Thus, while MRgRT is resource-intensive, its treatment time requirements are in proportion to other motion-managed or oART approaches for similar anatomic sites.

Our results compare favorably to the few studies that have described MRgRT times on both 0.35 T and 1.5 T MR-linacs, especially in the context of our case mix (Table 2). Güngör et al. reported that 83.2% of oART fractions delivered to 154 patients on a 0.35 T MR-Linac were completed within 60 min across a variety of anatomic sites [15]. In comparison, our TIRT results are similar although across a substantially larger experience with more upper abdominal tumors. Our early oART TIRT of 82.0 min during the initial 2018–2019 period was notably longer than the 46 min median reported by Güngör et al., most likely reflecting our frequent use of breath-hold gating for abdominal SBRT. Over time, this gap closed: by 2023–2024, median oART TIRT fell to 45.0 min, remaining low at 47.0 min in 2025–2026, when 78.7% of oART fractions were completed within 60 min, even with a more complex, abdominally focused case mix. Weykamp et al. reported outcomes across >2000 fractions delivered to various sites on a 0.35 T MR-linac that included longer median treatment times for adaptive versus non-adaptive MRgRT (71 vs. 36 min; *p* < 0.01) [14]. Rogowski et al. reported that adaptive SBRT on a 0.35 T MR-linac for liver tumors required a median treatment duration of 53 min [17]. Our results compare favorably with those reported from several 1.5 T MR-Linac studies although cross-platform comparisons should be interpreted cautiously because device design, adaptation strategy, gating capability, disease-site distribution, and institutional workflow differ. Stanescu et al. reported outcomes of liver and pancreas SBRT delivered on a 1.5 T MR-linac using abdominal compression with average treatment times of ~67 min [39]. A large study of 1000 patients treated on a 1.5 T MR-linac reported a mean in-room time of 38 min, although this is not comparable to our institutional experience since most were treated without gating for prostate cancer whereas our prostate experience is very limited [40].

This study has several limitations including its retrospective design. Our findings may not be generalizable to centers that do not treat a high volume of upper abdominal adaptive SBRT cases with automatic beam gating on a 0.35 T MR-linac with the A3i upgrade. Treatment times were manually documented by RTTs during each fraction instead of being extracted from machine log files, introducing the possibility of errors in reported times. A small proportion of fractions had incomplete time data. Subcomponents of TAT including recontouring, reoptimization, and QA times were not consistently recorded across our 8-year experience and thus were not able to be analyzed. The A3i system upgrade occurred mid-study in July 2022, making it difficult to disentangle the contributions of technological improvement from team learning-curve effects, particularly as concurrent changes in case volume, disease-site mix, and staffing were not controlled for. Case mix also shifted independently of workflow in 2023, when medical-necessity-based selection during the vendor’s bankruptcy eliminated two long-duration cohorts: 15-fraction lung courses requiring complex adaptation and density overrides (90 min/fraction) and CNS cases with post-delivery multisequence imaging (T1, T2, T2-FLAIR) counted within TIRT (~90 min/fraction). This change may have contributed to the late-period reduction in treatment times. The non-linear improvement trajectory, including transient increases in treatment times during certain periods, likely reflects unmeasured variables such as shifts in case complexity and staff turnover. Finally, this analysis focused exclusively on treatment efficiency and did not evaluate dosimetric plan quality or clinical outcomes.

## 5. Conclusions

In summary, our findings demonstrate that gated MRgRT with oART can be delivered efficiently for complex mobile tumors. Future advances in hardware, software, and artificial intelligence automation are expected to further reduce treatment times while enhancing treatment quality.

## Figures and Tables

**Table 1:** Baseline demographics and treatment characteristics. Note: Abdominal compression was not mutually exclusive with breath-hold or free-breathing techniques. Abbreviation: (NOS) not otherwise specified; (SBRT) stereotactic body radiotherapy; (GTV) gross tumor volume; (CTV) clinical target volume; (PTV) planning target volume; (BED) biological effectiveness.

Characteristic	Overall	2018–2022 (N = 506 Treatment Courses)	2022–2026 (N = 697 Treatment Courses)	*p* Value
	N (range or %)			
Median Age (Years)	69 (19–94)	69.0 (19.0–94.0)	69.0 (19.0–94.0)	0.379
Sex				
Female	552 (45.9)	225 (44.5%)	327 (46.9%)	0.400
Male	651 (54.1)	281 (55.5%)	370 (53.1%)	0.400
ECOG Performance Status				
0	375 (31.2)	205 (40.5%)	170 (24.4%)	<0.001
1	745 (61.9)	247 (48.8%)	498 (71.4%)	<0.001
2	69 (5.7)	44 (8.7%)	25 (3.6%)	<0.001
3	14 (1.2)	10 (2.0%)	4 (0.6%)	0.025
Median Body Mass Index (kg/m^2^)	26.1 (12.3–50.6)	26.2 (12.3–47.4)	26.0 (15.3–50.6)	0.430
Disease Site Fractions				
Pancreas	2232 (29.1%)	806 (20.4%)	1426 (38.4%)	<0.001
Thorax	1579 (20.6%)	1002 (25.4%)	577 (15.5%)	<0.001
Abdominal NOS	1031 (13.5%)	691 (17.5%)	340 (9.1%)	<0.001
Abdominopelvic Lymph Node	998 (13.0%)	438 (11.1%)	560 (15.1%)	<0.001
Liver	650 (8.5%)	277 (7.0%)	373 (10.0%)	<0.001
Adrenal	376 (4.9%)	228 (5.8%)	148 (4.0%)	<0.001
Pelvic NOS	341 (4.4%)	237 (6.0%)	104 (2.8%)	<0.001
Brain	145 (1.9%)	0 (0.0%)	145 (3.9%)	<0.001
Head and Neck	127 (1.7%)	104 (2.6%)	23 (0.6%)	<0.001
Prostate	102 (1.3%)	86 (2.2%)	16 (0.4%)	<0.001
Bone–Nonspine	48 (0.6%)	43 (1.1%)	5 (0.1%)	<0.001
Bone–Spine	36 (0.5%)	36 (0.9%)	0 (0.0%)	<0.001
Median Dose (Gy)	50.0 (16.0–76.0)	50.0 (16.0–76.0)	50.0 (20.0–76.0)	0.402
Median Fraction Numbers	5.0 (1.0–36.0)	5.0 (1.0–34.0)	5.0 (1.0–36.0)	<0.001
Median BED_10_ (Gy10)	100.0 (28.0–200.0)	94.3 (28.0–157.5)	100.0 (34.0–200.0)	<0.001
Median Treatment Course Duration (Days)	7.0 (1.0–65.0)	8.0 (1.0–65.0)	7.0 (1.0–51.0)	<0.001
Median GTV (cc)	18.8 (0.3–1330.6)	20.6 (0.6–1062.0)	17.6 (0.3–1330.6)	0.015
Median CTV (cc)	80.6 (0.7–1596.0)	58.4 (1.6–1450.6)	107.3 (0.7–1596.0)	0.002
Median PTV (cc)	213.8 (8.1–1809.1)	133.9 (12.0–1809.1)	233.3 (8.1–1004.9)	<0.001
Delivered Fractions				
Total	7665	3948	3717	
Online Adaptive	4277 (55.8%)	1297 (32.9%)	2980 (80.2%)	<0.001
Non-adaptive	3388 (44.2%)	2651 (67.1%)	737 (19.8%)	<0.001
Fractionation				
SBRT	4985 (65.0%)	1972 (49.9%)	3013 (81.1%)	<0.001
Conventional Fractionation	1440 (18.8%)	1123 (28.4%)	317 (8.5%)	<0.001
Moderate	1136 (14.8%)	749 (19.0%)	387 (10.4%)	<0.001
Palliative	104 (1.3%)	104 (2.7%)	0 (0.0%)	<0.001
Elective Coverage				
Yes	5347 (69.8%)	2440 (61.8%)	2907 (78.2%)	<0.001
No	2318 (30.2%)	1508 (38.2%)	810 (21.8%)	<0.001
Motion Management				
Breath Hold	6297 (82.2%)	2996 (75.9%)	3301 (88.8%)	<0.001
Free Breathing	1368 (17.8%)	952 (24.1%)	416 (11.2%)	<0.001
Abdominal Compression	444 (5.8%)	132 (3.3%)	312 (8.4%)	<0.001

**Table 2:** Summary of studies describing magnetic resonance-guided radiation therapy treatment times. NR indicates data not reported in the original study. The arrow indicates the change from the early to the later study period. Abbreviations: (GTV) gross tumor volume; (CTV) clinical target volume; (PTV) planning target volume; (IQR) interquartile range; (LN) lymph nodes.

Study	Patients/ Fractions	MR Platform	Treatment Sites	Target Volume Size	oART Utilization	oART TIRT (min)	Non-oART TIRT (min)
Herrera et al. (Current Study)	1026/7665	0.35 T	Pancreas (35.7%) Thorax (16.9%) Abdominal/pelvic LN (15.0%) Liver (11.5%) Adrenal (6.9%) Prostate (1.5%) Other (12.5%)	Median GTV: 18.8 cc; CTV: 80.6 cc; PTV: 213.8 cc	68.5% of courses 55.8% of fractions	Median 53.0 across all fractions 67.0 (2018–2022) → 48.0 (2022–2026)	Median 34.0 (IQR, 28.0–44.0) 35.0 (2018–2022) → 34.0 (2022–2026)
Weykamp et al. (2026) [14]	231/2020	0.35 T	LN (28.5%) Liver (19.4%) Lung (19.4%) Bone (13.9%) Adrenal (5.5%) Soft tissue (4.8%) Prostate (0.6%) Other (7.9%)	Median GTV: 10.7 mL; CTV: 19.9 mL; PTV: 36.4 mL	43.7% of courses	Median 70.9	Median 36.0
Votta et al. (2024) [16]	56/254	0.35 T	Nodes (21.4%) Rectum (21.4%) Lung (19.6%) Pancreas (14.3) Liver (12.5%) Other (5.4%) Adrenal (3.6%) Prostate (1.8%)	NR	70.9% of fractions	Median 33.4	Median 18.5
Güngör et al. (2021) [15]	154/962	0.35 T	Abdominal (46.8%) Pelvic (39.6%) Thoracic (21.4%)	Median GTV: 166 cm^3^, PTV: 166 cm^3^	80.4% of fractions	Median 46.0	NR
Rogowski et al. (2021) [17]	11/15	0.35 T	Liver (100%)	Median GTV: 16.5 cm^3^ PTV: 39.1 cm^3^	98% of fractions	Median 53.0	NR
Chen et al. (2026) [41]	90/250	1.5 T	Prostate (100%)	NR	100.0% of fractions	Median 67.6	NR
De-Colle et al. (2025) [40]	1000/9076	1.5 T	Prostate (72.0%) LN (16.0%) Bone (3.8%) Pancreas (1.9%) Liver (1.1%) Brain (1.0%) Adrenal (0.8%) Lung (0.8%) Other (2.5%)	NR	80.8% of fractions	Mean 38.0	NR
Lee et al. (2023) [42]	26/126	1.5 T	Pancreas (73.1%), LN (15.4%), Adrenal gland (11.5%)	Mean GTV: 36.6 mL; PTV 74.9 mL	61.1% of fractions	Mean 79.0	Mean 55.0
Stanescu et al. (2022) [39]	16/65	1.5 T	Liver (68.8%), Pancreas (3.21%)	NR	100% of fractions	Median Liver: 67.5 Pancreas: 80.0	NR
Turkkan et al. (2022) [43]	14/375	1.5 T	Prostate (100%)	NR	99.2% of fractions	Median 46.0	NR
Intven et al. (2021) [44]	43/204	1.5 T	Rectum (100%)	Mean CTV: 153 cc; PTV: 815 cc	100% of fractions	Median 48.0	NR
Bertelsen et al. (2019) [45]	19/176	1.5 T	Prostate (35.0%) Bone (30.0%) Soft tissue (20.0%) Ovaries (15.0%)	NR	28.0% of fractions	Median 42.0	Median 26.0

## Data Availability

Research data are stored in an institutional repository and will be made available upon request to the corresponding authors.

## Supplementary Materials

The following supporting information can be downloaded at: https://www.mdpi.com/article/10.3390/cancers18172814/s1, Figure S1: Violin plot of total in-room time for single-fraction stereotactic body radiation therapy according to anatomic site. Other disease sites are celiac plexus, kidney, and pelvic bone–spine metastases. Panel B: single-fraction treatment time metrics comparing online adaptive to non-adaptive; Figure S2: Median multi-fraction treatment times for the early (2018–2022) and late (2022–2026) study eras. Panel A shows total in-room time by workflow type, and Panel B shows online adaptive workflow components; Table S1: Treatment time metrics for online adaptive pancreas vs. non-pancreas cases by annual study period; Table S2: Treatment time metrics for online adaptive and non-adaptive fractions by annual study period; Table S3: Complete distribution of treatment characteristics and metric times by disease site.

## Abbreviations

The following abbreviations are used in this manuscript:

BED	Biological Effectiveness
CTV	Clinical tumor volume
GTV	Gross tumor volume
IMRT	Intensity-modulated radiation therapy
IQR	Interquartile range
LN	Lymph nodes
MLC	Multi-leaf collimator
MP	Medical physicist
MRgRT	Magnetic resonance-guided radiation therapy
NR	Not reported
oART	Online adaptive radiation therapy
PTV	Planning target volume
RO	Radiation Oncologist
RT	Radiation therapy
RTT	Radiation therapist
SBRT	Stereotactic body radiation therapy
TAT	Total adaptive time
TDT	Total delivery time
TIRT	Total in-room time
TrueFISP	True fast imaging with steady-state precision
