# Supplementary material for: Longitudinal Improvements in Treatment Delivery Efficiency for MR-Guided Radiation Therapy: An 8-Year Single-Institution Experience
Source: Cancers (Basel). 2026 Aug 31;18(17):2814. doi: 10.3390/cancers18172814 (PMC13565272; doi:10.3390/cancers18172814)
Supplement: Supplementary file 1 [file cancers-18-02814-s001.zip › cancers-4513353-supplementary.pdf]

cancers

MDPI

Supplemental Material

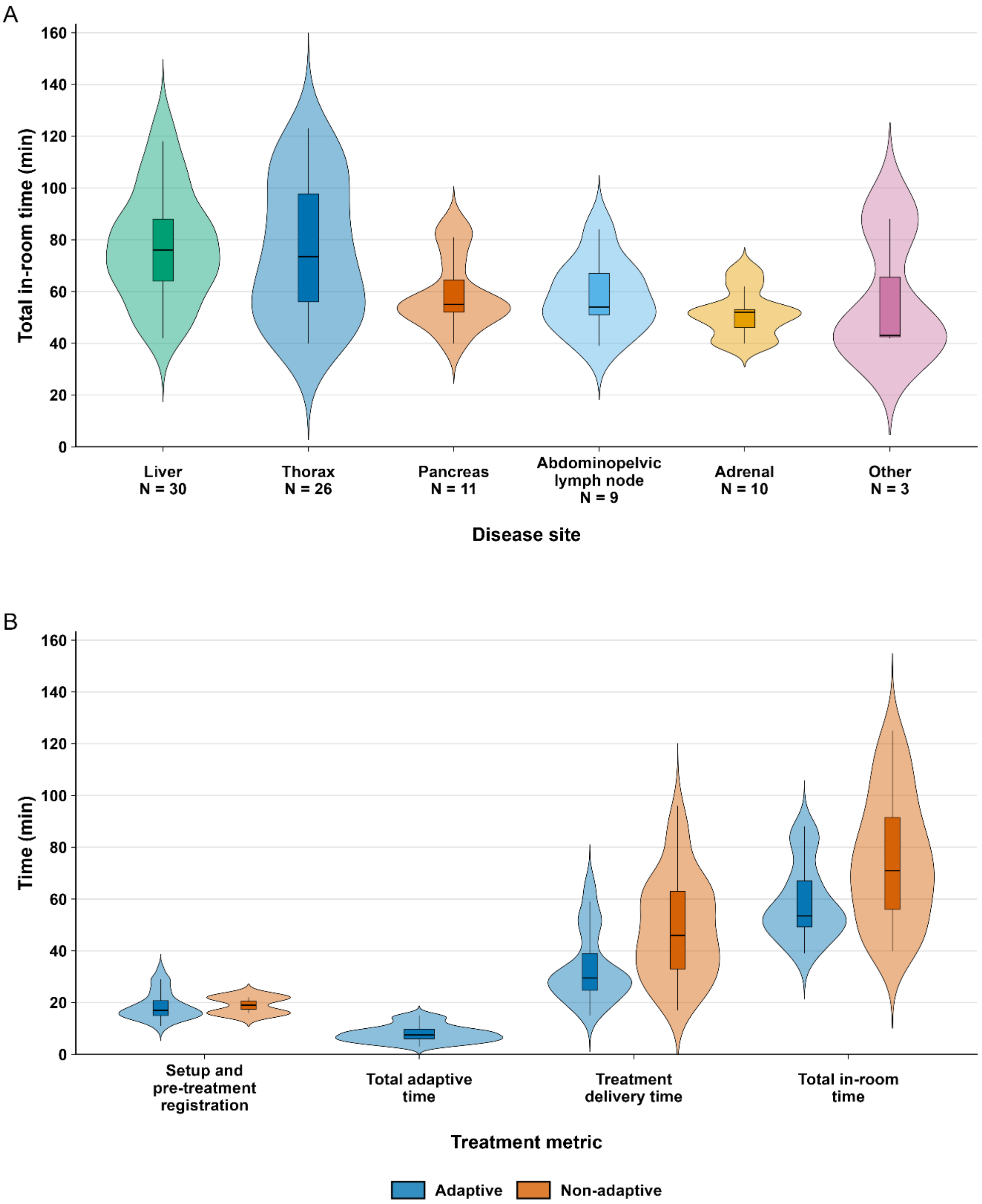

Figure S1. Violin plot of total in-room time for single-fraction stereotactic body radiation therapy according to anatomic site. Other disease sites are celiac plexus, kidney, and pelvic bone–spine metastases. Panel B: single-fraction treatment time metrics comparing online adaptive to non-adaptive.

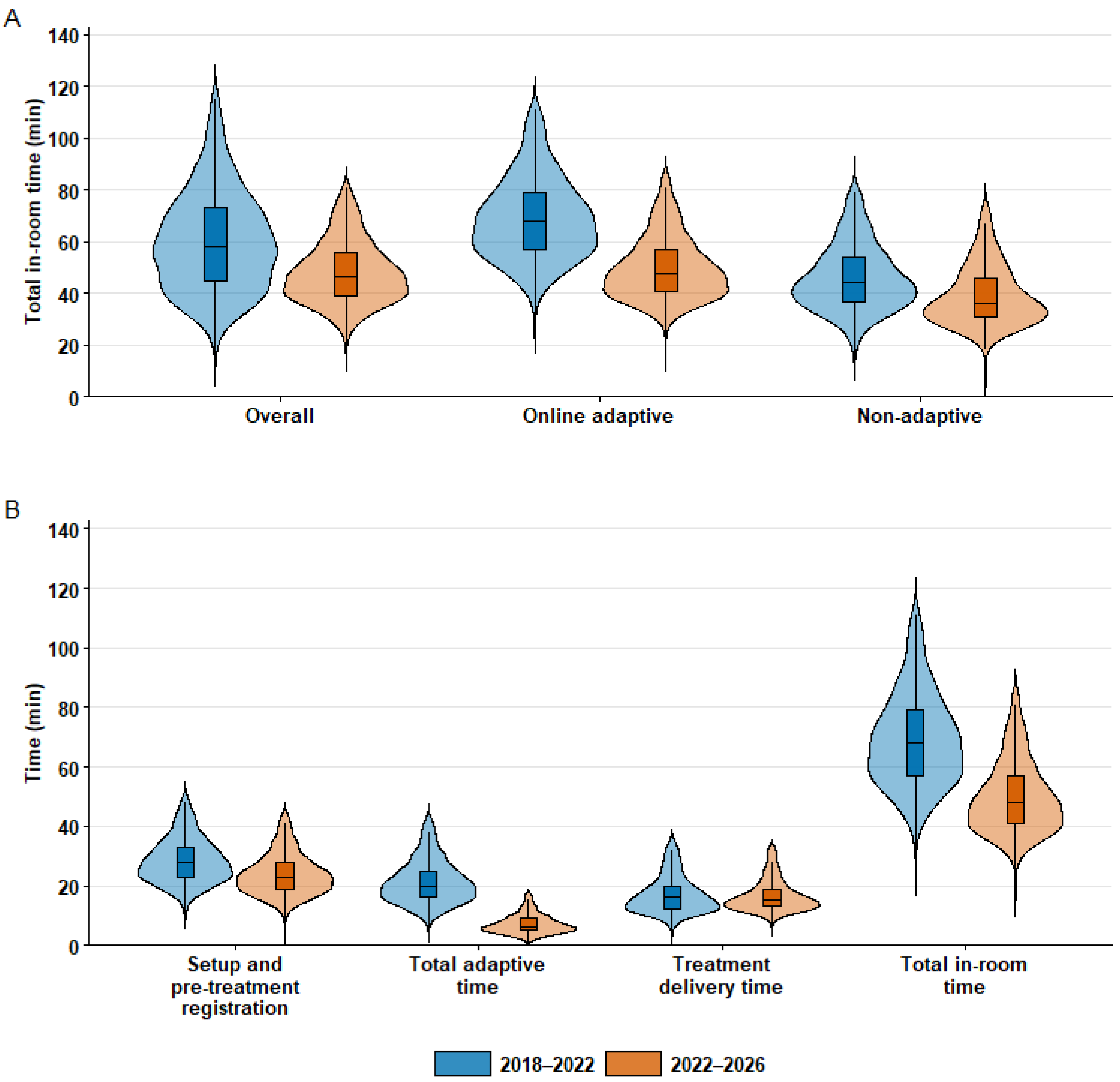

Figure S2. Median multi-fraction treatment times for the early (2018-2022) and late (2022-2026) study eras. Panel A shows total in-room time by workflow type, and Panel B shows online adaptive workflow components.

Table S1. Treatment time metrics for online adaptive pancreas vs non-pancreas cases by annual study period.

Study period	Treatment metric	Pancreas	Non-pancreas	P value
		median (IQR), min		
2018-2019	Setup and pre-treatment registration	27.0 (22.0–33.0)	27.5 (21.5–33.2)	0.985
	Total adaptive time	32.0 (24.0–42.0)	28.0 (22.8–33.5)	0.020
	Total delivery time	19.5 (16.0–27.0)	17.0 (14.0–26.0)	0.084
	Total in-room time	85.0 (72.0–94.8)	75.0 (63.0–86.0)	0.006
2019-2020	Setup and pre-treatment registration	29.0 (24.0–37.0)	29.0 (24.0–35.0)	0.743
	Total adaptive time	22.0 (19.0–28.0)	22.0 (19.0–29.0)	0.799
	Total delivery time	19.0 (14.2–25.0)	14.0 (11.0–22.0)	<0.001
	Total in-room time	75.0 (61.0–87.8)	69.0 (60.0–82.0)	0.078
2020-2021	Setup and pre-treatment registration	26.0 (23.0–32.5)	27.0 (23.0–31.0)	0.810
	Total adaptive time	18.0 (15.0–23.0)	18.0 (16.0–23.0)	0.653
	Total delivery time	14.0 (12.0–17.0)	15.0 (12.0–20.0)	0.429
	Total in-room time	63.0 (54.0–73.5)	63.0 (55.0–72.0)	0.868
2021-2022	Setup and pre-treatment registration	29.0 (23.0–34.0)	27.0 (22.0–33.0)	0.020
	Total adaptive time	18.0 (14.0–23.0)	18.0 (14.0–23.0)	0.837
	Total delivery time	16.0 (13.0–21.0)	13.0 (11.0–17.0)	<0.001
	Total in-room time	65.5 (56.8–76.2)	62.0 (53.0–73.0)	0.009
2022-2023	Setup and pre-treatment registration	22.0 (18.0–28.0)	23.0 (19.0–29.0)	0.094
	Total adaptive time	9.0 (7.0–13.0)	10.0 (7.0–14.0)	0.507
	Total delivery time	15.0 (12.0–18.5)	14.0 (11.0–19.0)	0.011
	Total in-room time	49.0 (39.0–60.0)	50.0 (43.0–59.0)	0.320
2023-2024	Setup and pre-treatment registration	21.0 (17.0–25.8)	20.0 (17.0–24.0)	0.063
	Total adaptive time	6.0 (5.0–9.0)	7.0 (5.0–10.8)	0.108
	Total delivery time	16.0 (14.0–20.0)	15.0 (12.0–21.0)	0.040
	Total in-room time	46.0 (40.0–55.5)	45.0 (38.0–56.0)	0.278
2024-2025	Setup and pre-treatment registration	24.0 (21.0–29.0)	24.0 (19.2–32.0)	0.944
	Total adaptive time	7.0 (5.0–8.0)	8.0 (6.0–11.0)	<0.001
	Total delivery time	15.0 (13.0–19.0)	18.0 (13.0–25.0)	<0.001
	Total in-room time	48.0 (42.0–56.5)	51.0 (44.8–67.0)	<0.001
2025-2026	Setup and pre-treatment registration	22.5 (19.0–26.2)	25.0 (20.0–32.0)	<0.001
	Total adaptive time	5.0 (4.0–7.0)	6.0 (5.0–9.0)	<0.001
	Total delivery time	16.0 (14.0–20.5)	17.0 (13.0–24.0)	0.101
	Total in-room time	45.0 (40.0–53.0)	50.0 (42.0–64.0)	<0.001

Table S2. Treatment time metrics for online adaptive and non-adaptive fractions by annual study period.

Study period	Treatment metric	Non-adaptive	Online Adaptive	P value
			median (IQR), min	
2018-2019	Setup and pre-treatment registration	24.0 (20.0–30.0)	27.0 (22.0–33.0)	0.317
	Total adaptive time	—	31.0 (24.0–39.0)	—
	Total delivery time	11.0 (8.0–17.0)	19.0 (15.0–27.0)	<0.001
	Total in-room time	40.0 (32.0–52.0)	82.0 (69.5–92.5)	<0.001
2019-2020	Setup and pre-treatment registration	29.0 (21.2–35.8)	29.0 (24.0–36.0)	0.414
	Total adaptive time	—	22.0 (19.0–28.0)	—
	Total delivery time	10.0 (8.0–13.0)	17.0 (12.0–24.0)	<0.001
	Total in-room time	30.0 (26.0–39.0)	72.0 (60.0–84.5)	<0.001
2020-2021	Setup and pre-treatment registration	26.0 (20.0–32.5)	27.0 (23.0–32.0)	0.258
	Total adaptive time	—	18.0 (15.0–23.0)	—
	Total delivery time	11.0 (8.0–17.0)	15.0 (12.0–19.0)	<0.001
	Total in-room time	35.0 (27.0–44.8)	63.0 (54.0–73.0)	<0.001
2021-2022	Setup and pre-treatment registration	23.0 (21.0–28.0)	28.0 (23.0–33.0)	<0.001
	Total adaptive time	—	18.0 (14.0–23.0)	—
	Total delivery time	13.0 (11.0–17.0)	14.0 (12.0–19.0)	<0.001
	Total in-room time	35.0 (29.0–43.0)	63.0 (54.0–74.0)	<0.001
2022-2023	Setup and pre-treatment registration	19.5 (15.8–24.5)	22.0 (18.0–28.5)	0.074
	Total adaptive time	—	10.0 (7.0–14.0)	—
	Total delivery time	14.0 (11.0–17.0)	14.0 (12.0–19.0)	0.016
	Total in-room time	31.0 (27.0–38.0)	50.0 (42.0–59.2)	<0.001
2023-2024	Setup and pre-treatment registration	19.0 (17.0–21.0)	20.0 (17.0–25.0)	0.591
	Total adaptive time	—	7.0 (5.0–10.0)	—
	Total delivery time	18.0 (15.0–24.0)	15.0 (13.0–20.0)	<0.001
	Total in-room time	36.0 (32.0–46.0)	45.0 (39.0–56.0)	<0.001
2024-2025	Setup and pre-treatment registration	18.0 (18.0–18.0)	24.0 (20.0–30.0)	0.209
	Total adaptive time	—	7.0 (6.0–10.0)	—
	Total delivery time	24.0 (16.0–46.0)	16.0 (13.0–21.0)	<0.001
	Total in-room time	48.5 (34.0–66.5)	50.0 (43.0–60.0)	0.161
2025-2026	Setup and pre-treatment registration	21.5 (17.5–25.8)	24.0 (19.0–29.0)	0.451
	Total adaptive time	—	6.0 (4.0–8.0)	—
	Total delivery time	17.0 (14.0–22.0)	16.0 (14.0–22.0)	0.349
	Total in-room time	34.0 (29.8–43.2)	47.0 (41.0–57.0)	<0.001

Table S3. Complete distribution of treatment characteristics and metric times by disease site.

Disease site	No. of treatment courses	No. of delivered fractions	Online Adaptive, fractions	Non-Adaptive, fractions	Total dose (Gy)	No. fractions	BED10 (Gy)	oART TIRT	Non-oART TIRT
	No, (%)				*Median (range)*			*Median (IQR)*	
Pancreas	430 (35.7%)	2232 (29.1%)	1991 (89.2%)	241 (10.8%)	50.0 (20.0–76.0)	5.0 (1.0–30.0)	100.0 (37.5–120.0)	52.0 (43.0–66.0)	33.0 (26.0–46.2)
Thorax	203 (16.9%)	1579 (20.6%)	556 (35.2%)	1023 (64.8%)	50.0 (24.0–60.0)	5.0 (1.0–30.0)	100.0 (36.0–149.6)	51.5 (43.0–63.0)	38.0 (32.0–48.0)
Abdominal or pelvic lymph node	181 (15.0%)	998 (13.0%)	787 (78.9%)	211 (21.1%)	42.0 (25.0–60.0)	5.0 (1.0–34.0)	86.4 (37.5–120.0)	53.0 (43.0-66.0)	32.0 (26.0-36.0)
Liver	138 (11.5%)	650 (8.5%)	265 (40.8%)	385 (59.2%)	50.0 (30.0–60.0)	5.0 (1.0–28.0)	100.0 (48.0–200.0)	55.0 (46.0-70.0)	39.0 (31.0-51.0)
Abdominal NOS	93 (7.7%)	1031 (13.5%)	261 (25.3%)	770 (74.7%)	42.0 (24.0–63.0)	6.0 (1.0–30.0)	72.0 (28.8–100.8)	55.5 (45.0-69.0)	30.0 (25.0-36.0)
Adrenal	83 (6.9%)	376 (4.9%)	298 (79.3%)	78 (20.7%)	50.0 (25.0–60.0)	5.0 (1.0–6.0)	100.0 (48.0–149.6)	60.0 (49.0-72.0)	47.0 (40.2-56.0)
Pelvic NOS	26 (2.2%)	341 (4.4%)	98 (28.7%)	243 (71.3%)	40.0 (35.0–65.0)	6.0 (5.0–36.0)	65.4 (40.3–100.0)	53.0 (44.0-61.0)	29.0 (26.0-34.0)
Prostate	18 (1.5%)	102 (1.3%)	6 (5.9%)	96 (97.1%)	40.0 (29.0–40.0)	5.0 (5.0–16.0)	72.0 (34.3–72.0)	55.0 (50.2-64.2)	40.5 (34.8-51.0)
Head & Neck	10 (0.8%)	127 (1.7%)	10 (7.9%)	117 (92.1%)	40.0 (28.0–68.0)	7.5 (5.0–32.0)	72.0 (34.0–100.0)	44.0 (39.2-46.0)	26.0 (23.0-32.0)
Bone–Spine	9 (0.7%)	36 (0.5%)	5 (13.9%)	31 (86.1%)	27.0 (16.0–35.0)	5.0 (1.0–6.0)	48.0 (28.0–59.5)	65.0 (57.0-84.0)	46.0 (37.0-57.0)
Bone–Nonspine	6 (0.5%)	48 (0.6%)	0 (0.0%)	48 (100.0%)	32.5 (20.0–66.0)	5.0 (3.0–33.0)	59.8 (28.0–79.2)	—	38.0 (35.0-42.2)
Brain	6 (0.5%)	145 (100.0%)	0 (0.0%)	145 (100.0%)	60.0 (27.0–60.0)	30.0 (3.0–30.0)	72.0 (51.3–72.0)	—	34.0 (32.0-38.0)
